# Individualized targeting of the posterior parietal cortex with intermittent theta burst stimulation in anorexia nervosa: a randomized, double-blind, sham-controlled protocol

**DOI:** 10.3389/fnins.2025.1733280

**Published:** 2026-01-21

**Authors:** Valentina Meregalli, Sofia Gentili, Arianna Menardi, Antonino Vallesi, Daniele Olivo, Renzo Manara, Andrea Serino, Gaia Risso, Fabio Sambataro, Angela Favaro, Enrico Collantoni

**Affiliations:** 1 Department of Neuroscience, University of Padua, Padova, Italy; 2 Padua Neuroscience Center, University of Padua, Padova, Italy; 3 MySpace Lab and NeuroRehab Research Center, Service of University Neurorehabilitation (SUN), Lausanne University Hospital, Institution of Lavigny and University of Lausanne, Lausanne, Switzerland

**Keywords:** anorexia nervosa, neuromodulation, precision psychiatry, transcranial magnetic stimulation (TMS), treatment resistance

## Abstract

**Background:**

Anorexia nervosa (AN) is a severe psychiatric disorder frequently marked by poor treatment response and chronic progression toward rigid, restrictive behaviors. These features highlight the need for innovative strategies targeting core mechanisms and symptoms of the disorder. This feasibility trial evaluates individualized intermittent theta-burst stimulation (iTBS) of the posterior parietal cortex (PPC) as an adjunct to treatment-as-usual, with the goal of identifying patient-specific targets and paving the way for personalized neuromodulation in AN.

**Methods:**

Thirty-four adults with AN (≥18 years, illness ≥12 months, ≥1 prior unsuccessful treatment) will be randomized (1:1) to 20 sessions of real or sham iTBS over 4 weeks. Stimulation will be delivered with a MagPro system and a Cool B70 A/P active/sham coil; motor threshold will be estimated with the Stochastic Approximator (SAMT) and re-checked weekly. Individualized stimulation target will be defined based on resting state functional magnetic resonance imaging data to identify the cortical region within the PPC that is maximally functionally connected with the activity of two meta-analytically derived networks representing body representation and implicit behavioral tendencies. Coil placement will be neuronavigated on individual T1 magnetic resonance imaging (MRI). Assessments will take place at baseline (T0), post-treatment (T1), and 4-month follow-up (T2), covering clinical status (body mass index (BMI), psychopathology, and quality of life), experimental tasks (implicit body representation, peripersonal space, approach–avoidance tendencies toward food, interoceptive accuracy, and neurocognitive functioning), and MRI. Feasibility endpoints include recruitment, retention, completion, blinding, and tolerability. Ethics approval: 6168/EST/25; registration: NCT07106645.

**Discussion:**

This trial emphasizes individualized PPC targeting and rigorous motor threshold (MT) estimation to advance personalized neuromodulation in AN. Feasibility and effect-size data will guide a confirmatory randomized clinical trial, while mechanistic analyses will test whether targeted iTBS strengthens body-schema circuits and aligns neural changes with clinical improvement, supporting a precision-psychiatry approach to eating disorders.

**Trail registration:**

https://clinicaltrials.gov/study/NCT07106645, identifier NCT07106645.

## Graphical Abstract

**Figure:**
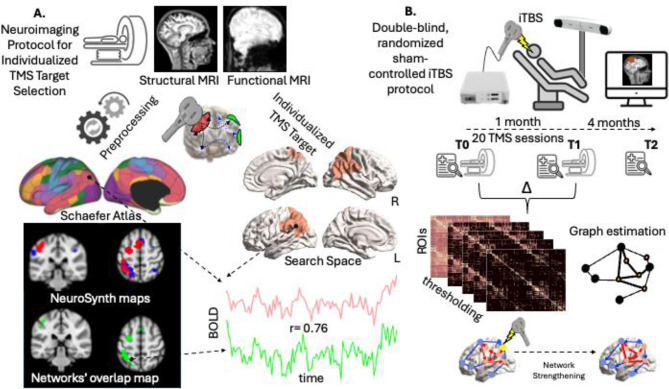
Graphical abstract summarizing individualized PPC target selection from resting-state fMRI and the randomized, double-blind, sham-controlled iTBS trial timeline in anorexia nervosa.

## Introduction

Anorexia nervosa (AN) is a severe and often enduring psychiatric disorder defined by a broad constellation of symptoms, among which two assume a central and defining role: the persistent restriction of caloric intake and profound disturbances in the experience of one's own body (American Psychiatric Association, 2013). Current therapeutic strategies rely on a multidisciplinary framework in which psychological interventions remain the cornerstone, while pharmacological and other biological treatments are mainly directed toward comorbid dimensions rather than the disorder's core pathology (Zipfel et al., 2015). Yet, despite these integrative approaches, a substantial proportion of patients fail to achieve full and sustained remission (Solmi et al., 2024). The combination of a protracted illness trajectory and repeated non-response to standard treatments often delineates forms of AN that become increasingly refractory to clinical intervention. From a cognitive perspective, this refractoriness is reflected in a progressive increase in rigidity and inflexibility while, on the behavioral plane, restrictive eating tends to crystallize into stereotyped, habit-like patterns (Treasure et al., 2020). Disturbances in body experience, in turn, manifest across multiple domains, ranging from dissatisfaction with body image to alterations in bodily perception, distortions of the body schema, and impairments in multisensory integration (Gadsby, 2017; Gentili et al., 2025). The trajectories through which these facets evolve over the course of illness remain incompletely understood. However, it is plausible that the more implicit dimensions of corporeal experience, such as the one related to body schema, remain especially resistant to psychotherapeutic interventions that rely on explicit, verbally mediated processes. These mechanisms contribute to the increasing independence of core symptoms from higher-order cognitive control, thereby reinforcing their chronicity, and treatment resistance.

Contemporary frameworks, including the Research Domain Criteria (RDoC) initiative and precision medicine paradigms, call for a reconceptualization of psychiatric disorders through neurobiological and neurocognitive perspectives, situated within dimensional and mechanistic models of psychopathology (Williams et al., 2024). Within this perspective, symptoms are no longer regarded as descriptive epiphenomena, but as emergent properties of dysfunctional neural circuits and altered cognitive processes (Buckholtz and Meyer-Lindenberg, 2012). The task of clinical neuroscience is therefore to delineate these mechanisms at behavioral, cognitive, and neurobiological levels, and to develop interventions capable of addressing them in a targeted and informed manner. Neuromodulation techniques such as transcranial magnetic stimulation (TMS) and transcranial direct current stimulation (tDCS) hold considerable promise in this respect, given their potential to selectively engage and modulate cortical networks implicated in the genesis and maintenance of symptoms (Liu and Wang, 2024). Their clinical impact rests on how thoroughly the underlying pathophysiological substrates are elucidated, characterized, and selectively targeted.

In AN, the application of neuromodulation remains at an early stage, particularly when contrasted with the more established use of these interventions in conditions such as major depressive disorder or obsessive–compulsive disorder (Rosson et al., 2022). To date, most studies have targeted the dorsolateral prefrontal cortex (DLPFC), based on its role in executive control, affect regulation, and top-down modulation of eating behavior. Preliminary evidence indicates that stimulation of the DLPFC can ameliorate core eating disorder psychopathology and comorbid affective symptoms, supporting its therapeutic relevance (Bahadori et al., 2025; Dalton et al., 2020). Nevertheless, the current evidence base is constrained by small sample sizes and methodological heterogeneity (Collantoni and Favaro, 2024). Moreover, while the DLPFC has been the most commonly targeted region, this choice has not been based on a direct link to the core symptoms of AN. In other clinical populations, selecting stimulation targets according to disorder-specific neurobiological knowledge has proven to be a promising and mechanistically informed strategy (Vinod et al., 2024). Therefore, a critical step for the advancement of neuromodulation in AN is to move beyond prefrontal targets and adopt alternative conceptual frameworks that can better capture the implicit dimensions of the disorder. Traditional interventions, both psychotherapeutic and pharmacological, have intrinsic limitations in this regard. Those psychotherapies that are grounded in explicit and reflective processes are poorly equipped to influence mechanisms that operate outside of conscious awareness, such as those shaping body representation or implicit learning. Conversely, pharmacological agents, while often effective in attenuating comorbid anxiety or depressive symptoms, show limited impact on the core features of AN, namely caloric restriction and disturbances in the experience of one's own body. This asymmetry underscores the need for novel interventions that can directly engage the neural substrates sustaining these implicit and more insidious symptoms.

Emerging theoretical accounts suggest that restrictive eating in AN cannot be fully explained as a purely goal-directed or intentionally maintained behavior, but may also involve automatic, stimulus-driven processes (Steinglass and Walsh, 2016). Such automatic responding is thought to arise from aberrant interactions between fronto-striatal and fronto-parietal systems, with posterior parietal regions contributing to the binding of sensory salience to action and to the automatization of behavioral routines (de Wit et al., 2012). This perspective positions restrictive eating not simply as a voluntary choice but as an entrenched behavioral pattern, resistant to change once consolidated.

With respect to body representation, it is well established that it is a multifaceted construct supported by a distributed network of cortical regions, including unisensory areas involved in body-related perception, motor and premotor regions supporting action-related and motor simulation processes, occipito-temporal regions involved in the visual and semantic representation of the body, and cortical regions implicated in self-referential processing within the default mode network, such as the posterior cingulate cortex (Azañón et al., 2016; Davey et al., 2016; Downing et al., 2001) rather than reflecting an impairment in a single body-related function, a substantial body of research has emphasized that disturbances in body experience in AN extend across multiple domains, including dissatisfaction with own body image, distortions in body size perception, and alterations in body schema and implicit body representations (Gadsby, 2017; Meregalli et al., 2022). Within this framework, regions that support the integration of different bodily information become particularly relevant. The posterior parietal cortex (PPC) represents a key associative hub within this distributed network, as it has extensive connections with motor and premotor cortices and integrates information coming from different sensory modalities to construct and update multisensory body representations, influencing body schema, body ownership, and the representation of the body in space (Chancel et al., 2022; Morasso et al., 2015). Consistently, several studies have suggested that, in patients with AN, alterations in PPC activity may be associated with distortions in body size estimation and broader disturbances in body representation (Mohr et al., 2010; Nico et al., 2010). Taken together, these perspectives converge on the PPC as a promising and underexplored target that bridges two symptom dimensions fundamental to AN: the rigidity of restrictive behaviors and the disturbances of body representation. The present protocol describes a randomized controlled trial (RCT) investigating the effects of real vs. sham stimulation over the PPC in patients with AN, delivered as an adjunct to treatment as usual (TAU). For each participant, the exact stimulation target will be individualized based on resting-state functional connectivity, with the aim of identifying the posterior parietal node showing the strongest connectivity with networks known to be involved in body representation and implicit behavioral tendencies. Participants will be randomly assigned to receive 20 sessions of either sham or real intermittent theta-burst stimulation (iTBS), a patterned TMS protocol thought to enhance cortical excitability in the target region and facilitate long-term neural plasticity (Huang et al., 2005). Importantly, the choice of the standard iTBS temporal pattern is primarily driven by its well-established plasticity-inducing properties, rather than by an assumption of a target-specific pathological theta rhythm in PPC. By directly engaging neural systems underlying the automatization of restrictive eating and the distorted body representation, this study aims to provide a mechanistically grounded therapeutic approach for patients with AN, while advancing the broader development of precision neuromodulation strategies in eating disorders. Outcome measures will be assessed at baseline, immediately after treatment, and at a 4-month follow-up. These will include: (1) a clinical evaluation conducted by an independent psychiatrist not involved in the study; (2) standardized clinical scales; (3) a neuroimaging protocol comprising both structural and functional measures; and (4) a battery of behavioral tasks.

The primary outcome of the study will be clinical improvement, assessed quantitatively through changes in BMI and scores on eating disorder psychopathology questionnaires, and qualitatively through the clinical judgment of the psychiatrist. We hypothesize that patients assigned to the active stimulation group will show greater BMI increase and greater reduction in psychopathology compared to the sham group.

Given that the stimulation protocol was specifically designed to target body representation and automatic tendencies toward food, secondary outcomes will focus on these aspects. Changes in body representation will be assessed using both an implicit task, which provides a measure of patients' perceived body boundaries, and an explicit task, in which patients will be asked to select the silhouette that best represents their own body. We hypothesize that, following the stimulation protocol, patients will show a more accurate representation of their body, selecting a silhouette closer to their actual body and demonstrating more precise boundary estimations.

Implicit behavioral tendencies toward food will be assessed using an approach–avoidance task. In a previous study, we observed that patients with AN exhibited a reduced tendency to approach food stimuli (Meregalli et al., 2024). We expect that, following the stimulation protocol, a more natural behavioral tendency will be restored, characterized by faster automatic approach of food stimuli.

## Methods

### Design

This study will adopt a double-blind, parallel-group, randomized controlled design with two arms. Patients with AN will be randomly assigned to undergo 20 consecutive weekday sessions of either real iTBS (treatment arm) or sham iTBS (control arm). Outcome assessments will take place at three time points: baseline (T0), immediately after the intervention (T1), and at a 4-month follow-up (T2). After completing the follow-up, participants allocated to the sham control arm will be given the option to receive real iTBS.

### Participants and recruitment

Participants will be recruited from the Eating Disorder Center of the Hospital of Padova, Italy. All eligible female patients followed at the center will be invited to take part in the study, as an adjunct to TAU, in agreement with their treating clinician. Recruitment will be limited to female patients due to the low number of male patients accessing the center and to minimize potential gender-related confounding factors.

Inclusion criteria will be: (1) current diagnosis of AN according to DSM-5 criteria; (2) age ≥18 years; (3) unsuccessful completion of at least one 3-month course of outpatient treatment; (4) illness duration of at least 1 year, and (5) female gender.

Exclusion criteria will be: (1) Presence of significant neurological comorbidities or severe/unstable systemic diseases, (2) Diastolic blood pressure < 60 mmHg or heart rate < 60 bpm, (3) diagnosis of psychosis or substance abuse disorders, (4) history of hypomanic/manic episodes, (5) Current suicidal ideation, (6) known contraindications to TMS as assessed with the TMS safety checklist (Rossi et al., 2021), including epilepsy, head trauma with loss of consciousness, severe/frequent headaches, cochlear implants, pacemakers, pregnancy, metallic implants, implanted neurostimulators; and (7) known contraindications to MRI, as assessed with the Hospital of Padova MRI safety checklist (Mod. Rev. 2, 16/05/2023), including presence of medical devices or metallic material, history of surgery or significant physical trauma, claustrophobia, pregnancy.

*A priori* power analysis conducted with G^*^Power (Faul et al., 2007) indicated that 28 participants are required to detect a significant group (2) × time (3) interaction assuming a medium effect size (Cohen's *f* = 0.25), statistical power of 1–β = 0.80, and significance level α = 0.05, with a correlation among repeated measures of *r* = 0.5. To account for potential dropouts, the sample size will be increased by 20%, resulting in a final target enrollment of 34 participants.

The study has been approved by the Ethics Committee of the Hospital of Padova (protocol number 6168/EST/25) and registered on ClinicalTrials.gov (registration number: NCT07106645). Written informed consent will be obtained from all participants prior to enrollment. Participants will be informed that their involvement is voluntary and that they may withdraw at any time without any consequences for their ongoing treatment.

### Intervention

#### Treatment as usual

In this study, iTBS is delivered as an add-on to treatment as usual (TAU) provided by a specialized eating-disorder service. TAU reflects a guideline-consistent, multidisciplinary and coordinated care pathway, and may include: (1) nutritional rehabilitation with dietetic assessment and monitoring, (2) regular medical monitoring of physical status and risk parameters, (3) eating-disorder-focused psychological support/psychotherapy, (4) psychoeducation, and (5) when indicated, family involvement/support. Pharmacological treatment may be part of TAU when clinically indicated. To reduce TAU-related confounding, the type and intensity of TAU will be kept as stable as clinically feasible during the 4-week stimulation period; any changes in treatment setting/intensity or medication will be prospectively documented. TAU intensity variables (e.g., outpatient vs. day-hospital setting, frequency of clinical/psychological/dietetic contacts, and medication changes) will be monitored throughout the study and considered in sensitivity analyses (and as covariates where appropriate).

#### Randomization and blinding procedure

Randomization will be implemented using patient codes provided by the coil manufacturer. The randomization procedure relies on the certified double-blind system integrated in the MagVenture MagPro platform for sham-controlled rTMS studies. Each participant will be assigned a unique code, corresponding to a pre-generated allocation to active or sham stimulation, which is entered into the TMS device at the beginning of each session. Participant codes are assigned at enrollment by a study member not involved in outcome assessments. Based on this code, the device automatically recognizes whether the participant receives real or sham stimulation, without revealing the stimulation condition to the operator or the participant. The coil is designed with two indistinguishable sides (active and sham), and the system instructs the operator only on whether the coil is in the correct position or needs to be rotated. Thus, the operator is aware only of coil positioning but remains blind to the actual stimulation condition. The randomization sequence is generated and managed externally to the study team through the device-coded procedure, ensuring full allocation concealment. Both participants and study personnel involved in treatment administration are therefore kept blinded to allocation, ensuring a double-blind design. At the end of the treatment, both participants and the operators who administered the stimulation will be asked to guess whether the participant received active or sham stimulation, as a measure of blinding integrity.

#### Motor threshold determination

Based on the participant's T1-weighted structural MRI, the Localite TMS Navigator system will be used to position the coil over the primary motor cortex (M1). Resting motor threshold (MT) will be determined using the SAMT (Stochastic Approximator of Motor Threshold) method, an adaptive algorithm designed to estimate MT efficiently and accurately. SAMT employs a digital control sequence with harmonic step adjustments, typically requiring around 20 pulses and achieving a median relative error of less than 1.5% (Wang and Peterchev, 2023; Wang et al., 2025). Electromyographic (EMG) recordings will be obtained from the first dorsal interosseous muscle to measure motor-evoked potentials (MEPs). At the beginning of the procedure, the operator will enter an initial stimulation intensity into the SAMT program. After each TMS pulse, the operator indicates whether a motor response has occurred—defined as an MEP of ≥50 μV. Based on this input, SAMT automatically adjusts the stimulation intensity for the next pulse, iteratively converging on the participant's MT in a controlled and efficient manner. To ensure safety and efficacy, the MT will be assessed weekly for each participant.

#### Localization of the stimulation target

**Figure 1:**
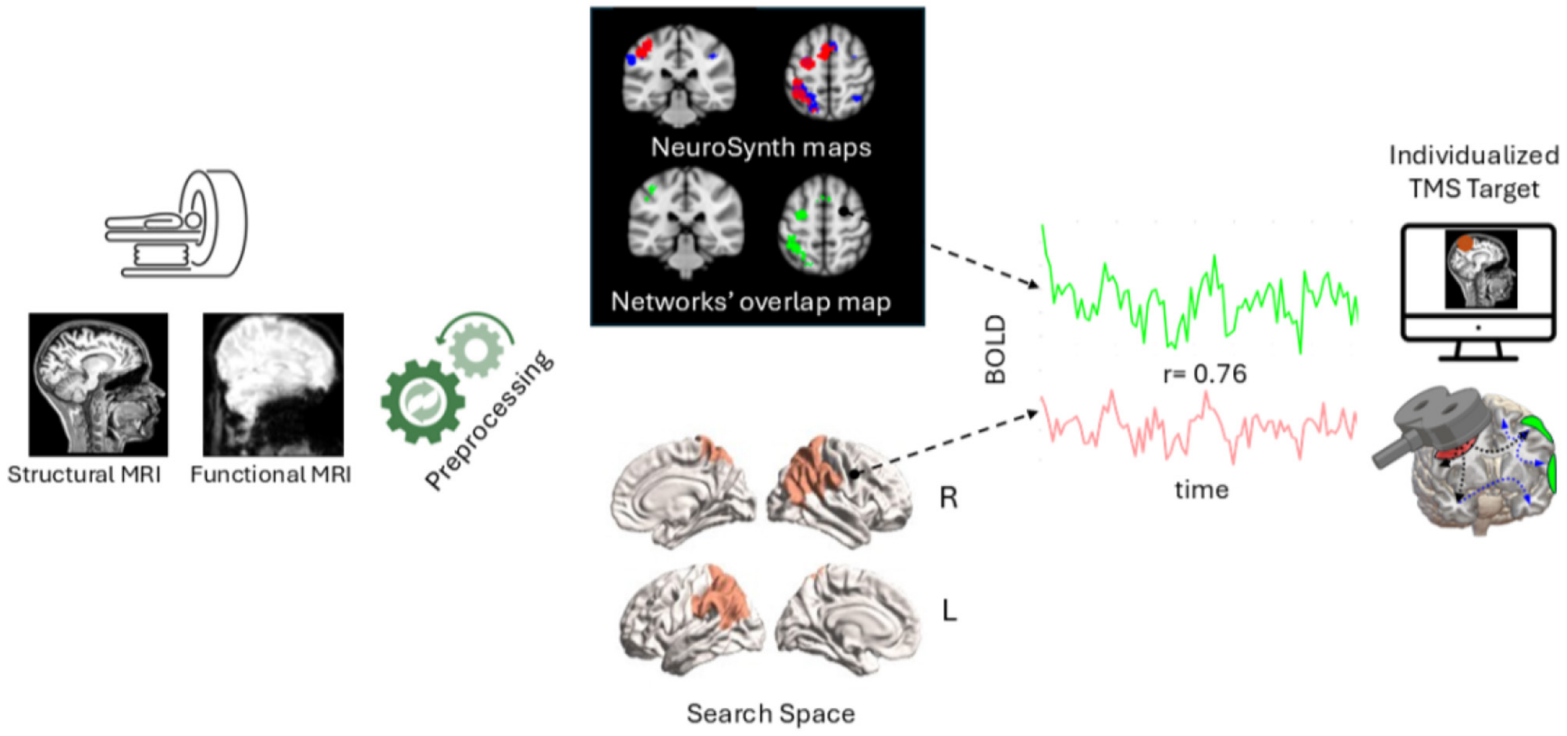
Graphical overview of the neuroimaging protocol for individualized TMS target selection.

The precise stimulation target will be individualized for each participant based on resting-state functional connectivity, with the aim of identifying the posterior parietal node showing the strongest connectivity with networks involved in implicit behavioral tendencies and body representation. These networks have been defined through a meta-analysis conducted using the Neurosynth platform (Kent et al., 2026), employing the following search terms: (1) “affordance” OR “habit” OR “motor program” and (2) “body representation” OR “body schema” OR “peripersonal” OR “multisensory integration” OR “embodiment.” In more detail, the Neurosynth-derived maps have been first thresholded to retain only the voxels with assigned values >2 standard deviations, hence ensuring that only regions that have been consistently reported across studies wilinl be retained. To further define a more precise target, the thresholded maps related to *behavioral tendencies-* and *body*- specific activations have been merged, binarized and only the voxels in overlap have been used to define the final target. The resulting Neurosynth-derived network primarily involved left-lateralized sensorimotor and parietal regions, with additional contributions from frontal, occipital and temporal cortices. Specifically, spatial overlap with the AAL3 atlas indicated involvement of the left precentral and postcentral gyri, bilateral supplementary motor area, left superior and inferior parietal lobules, left superior frontal gyrus, left middle occipital cortex, and left middle temporal gyrus (see Supplementary Table S1 for voxel-wise overlap). This resultant map will be co-registered to the individual functional neuroimaging space and used to extract its average blood oxygen level dependent (BOLD) signal over time (i.e., for the entirety of the functional neuroimaging scan acquisition). Next, we will define a search space consisting of 37 bilateral posterior parietal regions derived from the Schaefer 200 parcels atlas (Schaefer et al., 2018), which will also be coregistered to the individual functional neuroimaging space (for more details, see MRI protocol section). The individualized TMS target will be chosen as the region that is maximally functionally connected with the networks' overlap map based on the Pearson's correlation between their timeseries. To ensure biological meaningfulness and consistency of individualized targeting, only regions showing at least moderate functional connectivity with the overlap network will be considered eligible targets (*r* > 0.30). Of note, in consideration of the fact that the TMS pulse cannot reach deep cortical structures, no medial regions will be considered in the search space. The methodological overflow is depicted in Figure 1.

Once the individual TMS target is identified, its Montreal Neurological Institute (standard space/template) (MNI) coordinates will be communicated to the researcher in charge of delivering the stimulation, which will happen within a week from the neuroimaging acquisition. At the time of stimulation, coil positioning on the scalp will then be guided using the Localite TMS Navigator system, based on the participant's T1-weighted structural MRI and three skull landmarks (inion and the outer canthi of both eyes), allowing accurate placement of the coil over the individualized target. The coil position will subsequently be marked on the participant's cap, so that the full localization procedure needs to be performed only during the first session.

#### Stimulation sessions

TMS will be delivered using a MagPro stimulator (MagVenture, Denmark) equipped with a Cool B70 A/P coil, which allows for active stimulation on one side and integrated sham functionality on the opposite side, ensuring a reliable double-sham protocol.

The procedure and stimulation will be identical for participants in both the active and control groups, with the only difference being that in the sham condition the coil is oriented so as not to stimulate the brain, as described above.

All participants will receive 20 sessions of TMS over 20 consecutive weekdays. Except for the first session, which will be longer due to the requirements of neuronavigation, the remaining sessions are expected to last approximately 20–30 min, including preparation time, 3 min of stimulation, and debriefing. The stimulation protocol will follow a standard iTBS paradigm, consisting of bursts of three pulses at 50 Hz, repeated at 5 Hz for a total duration of 3 min, delivered at 100% of the participant's resting motor threshold (Huang et al., 2011). This temporal pattern was selected for its documented capacity to induce durable, plasticity-like modulation of cortical excitability, in line with established theta-burst stimulation frameworks, and not to directly entrain region-specific theta oscillations.

#### Safety monitoring and side effects

All study procedures and stimulation parameters will follow the most recent international safety and application guidelines for TMS (Rossi et al., 2021). For each participant, a case report form will be maintained to document session attendance, protocol adherence, and the occurrence of any side effects or adverse events based on predefined criteria. Minor adverse effects (e.g., transient headache) will not necessitate withdrawal from the study, although participants will retain the right to discontinue treatment at any time. In contrast, TMS will be immediately interrupted if a serious adverse event occurs (e.g., an epileptic seizure). After the first and the last stimulation session, participants will also complete the TMSens_Q, a standardized questionnaire designed to record any sensations or adverse effects experienced during or after TMS, in order to systematically monitor tolerability and safety (Giustiniani et al., 2022). In addition to minor side effects, potential longer-lasting effects on visuospatial attention due to PPC stimulation will be monitored with pre- and post-stimulation neurocognitive assessment.

### Outcome measures

**Figure 2:**
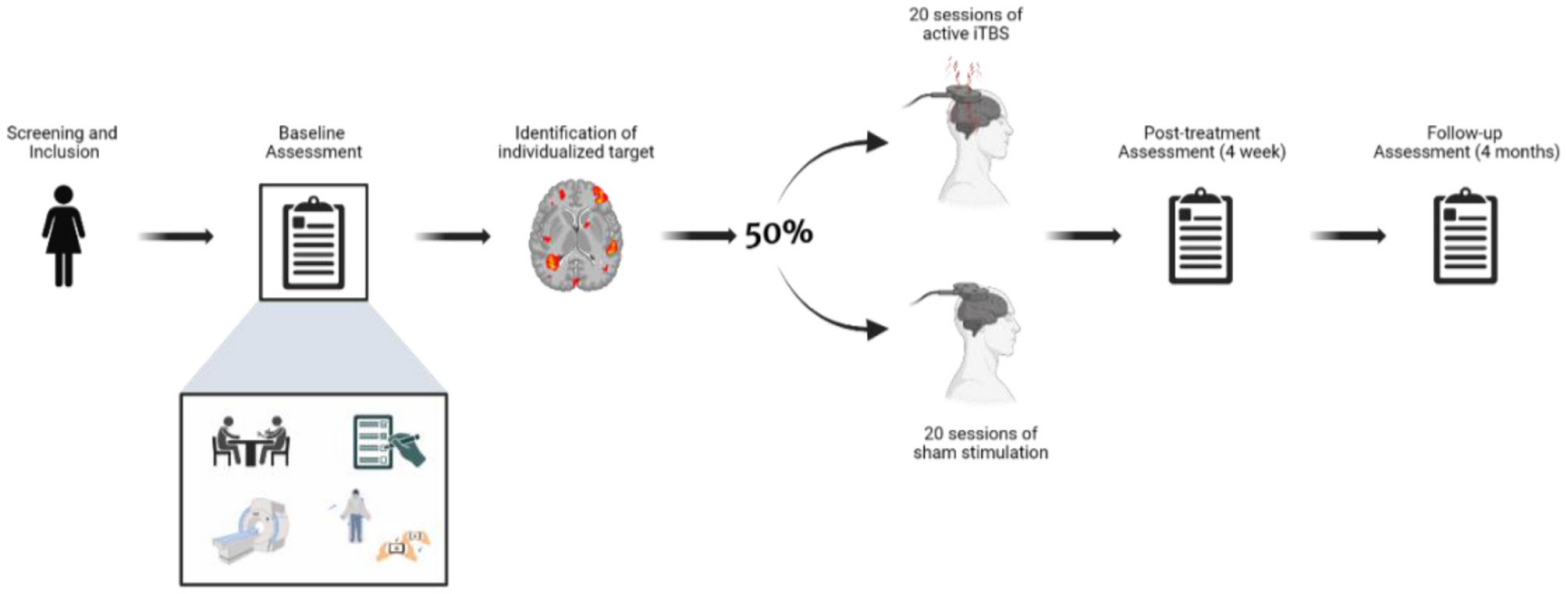
Schematic representation of the study protocol. Following baseline assessment, participants will be randomized to active or sham TMS and complete 20 stimulation sessions. Post-treatment and follow-up assessments will replicate baseline measures (with the exception of MRI at follow-up).

In the week prior to the start of treatment, all participants will undergo a baseline assessment consisting of: (1) an independent clinical evaluation conducted by a psychiatrist not involved in the study; (2) self-administered clinical scales; (3) an MRI scan including both structural and functional sequences; and (4) an assessment battery comprising behavioral and neurocognitive tasks designed to evaluate implicit body representation, food-related processing, and broader cognitive functions. The post-treatment assessment will take place in the week immediately following the final TMS session and will include the same components as the baseline assessment. Finally, a follow-up evaluation will be conducted 4 months after the post-treatment assessment, repeating all baseline and post-treatment measures, with the exception of the MRI scan. See Figure 2 for a graphical description of the procedure.

#### Clinical evaluation

At all three time points, an independent psychiatrist will conduct a semi-structured interview, systematically addressing expectations and concerns about TMS, anxious and depressive symptoms, daily functioning and quality of life, ruminative and repetitive thoughts, and core eating disorders psychopathology. Additional domains such as obsessive-compulsive symptoms, impulsivity levels, and levels of insight will be explored if clinically relevant. This qualitative assessment will complement standardized scales in evaluating symptom change and treatment acceptability. From this interview, the following outcome measures will be extracted: body weight and BMI, presence or return of menses, and any modification of the psychopharmacological treatment. In addition, the psychiatrist will record changes in eating behaviors (restriction, binge episodes, purging, and excessive compensatory exercise), levels of preoccupation with weight and body image, mood and anxiety severity, and global clinical impression (CGI-S/CGI-I). These measures will provide complementary indicators of treatment response and acceptability alongside standardized scales.

#### Self-administered scales

At all three time points, patients will complete the following self-administered scales using the online platform Qualtrics: (1) the *Eating Disorder Examination Questionnaire* (Fairburn and Beglin, 1994), a 28-item measure assessing the severity of eating disorder psychopathology across the domains of restraint, eating concern, weight concern, and shape concern; (2) the *State-Trait Anxiety Inventory* (Spielberger, 1983), a 40-item questionnaire that assesses both state and trait anxiety symptoms; (3) the *Depression Anxiety Stress Scales* (Lovibond and Lovibond, 1996), a 21-item instrument assessing symptoms of depression, anxiety, and stress; (4) the *Multidimensional Assessment of Interoceptive Awareness* (Mehling et al., 2012), a 32-item scale evaluating multiple facets of interoceptive body awareness (e.g., noticing, attention regulation, and emotional awareness; 5) the *UPPS-P Impulsive Behavior Scale* (Whiteside and Lynam, 2012), which measures five distinct dimensions of impulsivity: negative urgency, lack of premeditation, lack of perseverance, sensation seeking, and positive urgency; the (6) *Body Shape Questionnaire* (Cooper et al., 1987), a 34-item questionnaire developed to evaluate negative self-appraisal of body shape (e.g., the desire to lose weight, fear of gaining weight, and self-devaluation in relation to physical appearance); and (7) the *Weight Bias Internalization Scale* (Pearl and Puhl, 2014), an 11-item questionnaire developed to measure the degree of negative beliefs about one's weight or body size.

#### MRI protocol

Structural MRI data will be acquired on a 3T Philips Ingenia scanner (TR = 6.8 ms, TE = 3.1 ms, slice thickness = 1 mm, Flip Angle = 8°, 181 × 480 × 480 matrix, voxel size = 1 × 1 × 1 mm^3^). Preprocessing of the structural images will involve brain extraction and bias field correction by means of ANTs (Advanced Normalization Tools; antBrainExtraction and N4BiasFieldCorrection functions; Tustison et al., 2021). The FMRIB Software Library (FSL) FMRIB's Automated Segmentation Tool (FAST) algorithm will be used to perform tissue segmentation (Zhang et al., 2001), followed by MNI normalization by means of ANTs (Tustison et al., 2021). Functional MRI data will be acquired on a 3T Philips Ingenia scanner (TR = 800 ms, TE = 24.6 ms, flip angle = 60°, number of volumes = 800, voxel size = 2.78 × 2.78 × 3 mm, 96 × 96 × 56 matrix). Preprocessing of the functional images will involve motion correction by means of mcflirt (Jenkinson et al., 2002), followed by bias field correction by means of ANTS (Tustison et al., 2021). FMRIB's Linear Image Registration Tool (FLIRT) will be used to coregister each participant's functional and anatomical volume using 12 degrees of freedom and a boundary-based constriction relying on each individual white matter mask (Greve and Fischl, 2009). CompCor (Components based Noise Correction; Behzadi et al., 2007) and ART (Artifact Rejection Toolbox) functions will be further applied to perform scrubbing and remove additional noise components following the CONN functional connectivity toolbox (MATLAB-based) (CONN) denoising pipelines (Nieto-Castanon, 2020). A gaussian smoothing kernel with a full width at half maximum of 6 mm will be applied as a last step to further improve the signal to noise ratio. Finally, the Schaefer 200 functional parcellation (Schaefer et al., 2018) will be coregistered to the individual EPI space by applying the transformation matrix of the MNI to structural-functional coregistration. The same procedure will be applied to bring the networks' overlap map from its MNI space to the individual space. Functional timeseries will then be extracted from both the atlas and the networks' overlap map, representing the fluctuations of the BOLD signal across all volumes of the acquisition (*n* = 800).

The same acquisition and preprocessing steps will be repeated for the post-treatment acquisition.

#### Behavioral and neurocognitive tasks

At all time-points participants will undergo an assessment battery including the following behavioral and neurocognitive tasks:

(1) *Virtual reality task to assess implicit body representation*. In this task, participants see in virtual reality a green dot approaching them from different orientations and at varying heights. They are instructed to press a button at the exact moment they perceive the stimulus to have “touched” their body. This procedure allows for the construction of a perceptual map of body boundaries in the absence of direct visual or tactile feedback, thereby providing an implicit measure of body size distortion.
(2) *Figure rating scale*. Participants will be presented with a series of computer-generated silhouettes created using the MakeHuman software, representing a range of BMI from 12 to 38. They will be asked to select the silhouette that best corresponds to their perceived current body shape and the silhouette that represents their desired body shape. Two derived indices will be calculated: the difference between perceived BMI and actual BMI, providing an implicit measure of body size distortion, and the difference between desired BMI and perceived BMI, serving as an index of body dissatisfaction.
(3) *Peripersonal space task in the presence of food stimuli*. This task is an adaptation from an established paradigm for measuring peripersonal space (PPS; Serino, 2019). As in standard PPS tasks, participants are immersed in virtual reality and presented with visual stimuli approaching them. Simultaneously, a tactile stimulus is delivered to the participant, who is instructed to respond as quickly as possible by pressing a response button. The tactile stimulus is administered when the visual stimulus is at one of five possible distances from the participant. The underlying principle is that when the visual stimulus enters the PPS, the response to the tactile stimulus is facilitated, allowing reaction times to serve as an implicit measure of PPS extent. Given that PPS representation is known to be modulated by various factors, including the nature of the approaching stimulus, we will assess both the extent and shape of PPS in the presence of high-calorie food stimuli and neutral objects.
(4) *Approach-avoidance task toward food stimuli*. Implicit behavioral tendencies toward food stimuli will be assessed using a mobile version of the approach-avoidance task (Collantoni et al., 2023). In this task, participants are instructed to either approach images by pulling their phone toward themselves or avoid images by pushing it away. Stimuli include high-calorie foods, low-calorie foods, and neutral objects. Reaction times for each movement will be recorded, and an approach bias score will be calculated by subtracting avoidance reaction times from approach reaction times, providing an index of implicit motivational tendencies toward the different types of stimuli.
(5) *Heartbeat counting task*. Interoceptive accuracy will be assessed using the heartbeat counting task (Garfinkel et al., 2015). Participants are asked to silently count their own heartbeats over several fixed time intervals (e.g., 25, 35, 45 s) without manually checking their pulse. The counted heartbeats are then compared to the actual number of heartbeats recorded via a heart rate monitor, allowing calculation of an accuracy score that reflects the participant's ability to perceive internal bodily signals.
(6) *Trail making test*. The TMT (Reitan, 1958) will be used to assess cognitive flexibility. The task consists of two parts: Part A, in which participants connect numbered circles in ascending order as quickly as possible, primarily measuring processing speed; and Part B, in which participants alternate between numbers and letters in ascending/alphabetical order (1-A-2-B, etc.), requiring set-shifting and cognitive flexibility. The time to complete each part is recorded, and the difference or ratio between Part B and Part A is commonly used as a proxy of executive functioning and mental flexibility.
(7) *Rey-Osterrieth complex figure*. The ROCF (Osterrieth, 1944) will be administered to evaluate visuospatial abilities, with a specific focus on assessing central coherence during figure construction. The test consists in the copy of a complex geometric figure, followed by a recall trial of the same figure after a 3-min interval filled with non-interference tasks. The entire procedure will be video-recorded and conducted using the pen switching method. Scoring will be performed according to the Taylor method for *Accuracy* and Booth's criteria for the *Central Coherence Index* (Lezak et al., 2004). To minimize implicit learning that may occur with repeated administration of the same figure in longitudinal assessments, the Modified Taylor Complex Figure (MTCF), an equivalent form of the ROCF, will be used interchangeably in the pre-post treatment evaluation (Hubley and Jassal, 2006). Scoring of the MTCF will be conducted using the same standardized methods as for the ROCF, appropriately adapted.

The order of task presentation will be randomized across participants and sessions. Each assessment session is expected to last approximately 1.5 h.

### Analyses

#### Feasibility

Feasibility will be assessed through several indicators, including recruitment and retention rates, number and reasons for drop-outs, and adherence to the planned sessions. Acceptability and tolerability of the protocol will be evaluated based on participants' feedback, the presence of adverse events or side effects, as measured with the TMSens_Q, and overall satisfaction with the intervention as measures with a likert scale from 0 to 100.

#### Clinical, behavioral and cognitive outcomes

Analyses will be conducted according to the intention-to-treat principle. For each outcome, longitudinal changes will be evaluated using linear mixed-effects models (LMMs) with fixed factors of Group (active vs. sham), Time (baseline, post-treatment, 4-month follow-up), and their interaction (Group × Time). Random intercepts for participants will be included to account for within-subject correlations across repeated measurements. Effect sizes and 95% confidence intervals will be reported alongside p-values.

#### Functional connectivity modifications

In order to assess if behavioral modifications will be accompanied by an underlying change in the connectivity of the TMS target, we will statistically compare pre and post connectivity estimates by means of a 2 × 2 mixed Analyses of Variance (ANOVA), with the two factors representing respectively the Group (active vs. sham, between-subjects) and the Time (baseline vs. post-treatment, within-subject). The graph theory framework will be used to verify our hypothesis that the individualized stimulation protocol will result in enhanced connectivity between the parietal TMS target and the networks' overlap map (i.e., the conjunction between the neural regions involved in body schema representation and in implicit behavioral tendencies). In more detail, we expect the active group to show increased connectivity, i.e., a lower path length, in the post-treatment compared to the pre-treatment scan between these two sites, where the path length is measured as the inverse of the connectivity strength. At the same time, we expect that the broader network of regions involved in both body schema and implicit behaviors will show greater global efficiency, which reflects enhanced integration between distant nodes and is mathematically computed as the inverse of the averaged path lengths between all pairs of nodes, such as that short paths between all pairs of nodes translate in greater global efficiency of the overall network. We expect increased global efficiency as a result of the repeated activation of the targeted regions by means of the stimulation. Finally, in consideration of the fact that the TMS-induced effects on the functional connectivity can extend beyond the stimulation target (Beynel et al., 2020), we will address potential changes in the overall connectivity of the brain. In particular, we will test if the brain connectome might benefit from a functional re-arrangement that might lead to a concomitant increase in both global (every region can be linked by means of a short few paths) and local (regions cluster together into specialized modules) efficiency. This property is known as small worldness, which the literature suggests being decreased in AN patients compared to controls (Geisler et al., 2020). Small-worldness reflects hence the ratio between the average path length of a network and its clustering coefficient (i.e., a measure of the tendency of neighboring nodes to form triangular triples and thus to display high local efficiency).

## Discussion

The present protocol introduces an attempt to broaden the application of TMS in AN by moving beyond the prefrontal cortex, which has so far represented the predominant neuromodulation target (Bahadori et al., 2025). Here, stimulation is directed to the PPC with the aim of modulating neural systems involved in body representation and in the emergence of rigid, implicitly driven eating behaviors, two domains that are central to the disorder and often resistant to conventional treatments. Target localization is individualized on the basis of resting-state connectivity, identifying posterior parietal nodes with the strongest co-activation within circuits relevant to body representation and cue-triggered eating behaviors. This approach provides a neurobiologically informed rationale for intervention and aligns with the principles of precision psychiatry, by tailoring stimulation to networks relevant in the maintenance of specific symptom dimensions (Williams et al., 2024). The outcome assessment is designed to span multiple levels. Core clinical outcomes include body weight and BMI, menstrual status, and changes in eating disorder psychopathology. Broader psychopathological dimensions such as anxiety and depression are also systematically evaluated. In addition, the study places particular emphasis on implicit processes related to body representation and food-related behavior, domains that are less accessible through self-report measures. To this end, the protocol incorporates experimental paradigms delivered through virtual reality and mobile applications, allowing the capture of distortions in body representation and automatic responses that are rarely addressed in routine clinical assessments. Together, these multimodal measures aim to provide a more comprehensive picture of treatment effects, bridging clinical symptomatology with underlying cognitive processes and neural mechanisms.

This protocol also faces important challenges, both clinical and methodological, that deserve careful attention. First, adherence and engagement can be challenging in AN, where ambivalence for treatment and strong need for control are common. Qualitative accounts embedded in an rTMS feasibility program describe rTMS as acceptable yet time-consuming, with mixed reactions about “brain-directed” interventions and their impact on agency, underscoring the importance of careful consent, expectation management, and qualitative feedback throughout the trial (Dalton et al., 2022).

Second, the PPC is a novel and largely unexplored stimulation site in psychiatry, making it particularly important to monitor tolerability and carefully document potential side effects, including common discomforts such as headache or fatigue. We however expect that adverse effects such as pain or discomfort from nerve stimulation under the skin due to rTMS will be milder compared to the stimulation of frontal regions, which are more richly innervated.

Third, stakeholder attitudes may also influence feasibility. Qualitative studies indicate that patients, family members, and clinicians can all express reservations, though for different reasons. Family members often highlight concerns about autonomy, identity, or the perceived intrusiveness of brain stimulation, while clinicians sometimes emphasize uncertainties regarding efficacy, logistical demands, or the potential to disrupt therapeutic engagement. These perspectives suggest that transparent communication with both relatives and treating teams is essential, not only to sustain participation but also to contextualize possible benefits and risks within ongoing care (Dalton et al., 2021).

Fourth, methodological risks also warrant consideration. Achieving a convincing sham condition remains challenging in TMS trials, even with the use of advanced double-coil systems, as subtle differences in scalp sensations or auditory cues may compromise blinding (Duecker and Sack, 2015), with potential resentful demoralization that could jeopardize compliance, or compensatory rivalry that could hinder the detection of treatment effects (McCambridge et al., 2014). Moreover, the mere possibility of being randomized to the sham arm can reduce willingness to participate and heighten concerns about the treatment, particularly in a population where ambivalence toward intervention is already common. For these reasons, assessing blinding integrity and carefully documenting reasons for refusal or discontinuation will be crucial for interpreting trial outcomes and for informing the design of future studies.

In summary, this protocol combines a neurobiologically informed stimulation strategy with outcome measures that capture both explicit and implicit dimensions of AN, while addressing the practical and methodological challenges inherent to this population. The findings are expected to inform not only the feasibility of posterior parietal non-invasive stimulation in treating AN, but also its potential role within the broader landscape of precision interventions for eating disorders.
