# Supplementary material for: Individualized targeting of the posterior parietal cortex with intermittent theta burst stimulation in anorexia nervosa: a randomized, double-blind, sham-controlled protocol
Source: Front Neurosci. 2026 Jan 21;19:1733280. doi: 10.3389/fnins.2025.1733280 (PMC12868229; doi:10.3389/fnins.2025.1733280)
Supplement: Supplementary file 1 [file Table_1.docx]

| **AAL3 Region** | **Hemisphere** | **Number of overlapping voxels** |
| --- | --- | --- |
| Precentral gyrus | Left | 147 |
| Superior frontal gyrus | Left | 81 |
| Supplementary motor area | Left | 74 |
| Supplementary motor area | Right | 14 |
| Postcentral gyrus | Left | 60 |
| Superior parietal lobule | Left | 103 |
| Inferior parietal lobule | Left | 214 |
| Middle occipital gyrus | Left | 41 |
| Middle temporal gyrus | Left | 11 |

**Supplementary Table S1.** *Voxel-wise spatial overlap between the Neurosynth-derived network and anatomical regions defined by the Automated Anatomical Labeling atlas (AAL3).*
